# Arbovirus persistence in North-Western Europe: Are mosquitoes the only overwintering pathway?

**DOI:** 10.1016/j.onehlt.2022.100467

**Published:** 2022-12-01

**Authors:** Rody Blom, Maarten J.J. Schrama, Jeroen Spitzen, Babette F.M. Weller, Anne van der Linden, Reina S. Sikkema, Marion P.G. Koopmans, Constantianus J.M. Koenraadt

**Affiliations:** a Laboratory of Entomology, Plant Sciences Group, Wageningen University, Wageningen, the Netherlands; b Institute of Environmental Sciences, Leiden University, Leiden, the Netherlands; c Viroscience, Erasmus MC, Rotterdam, the Netherlands; d Centre for Avian Migration, Netherlands Institute of Ecology (NIOO-KNAW), Wageningen, the Netherlands

**Keywords:** Usutu virus, West Nile virus, Sindbis virus, *Culex pipiens*, Diapause

## Abstract

In some areas in temperate Europe, genomic analyses of mosquito-borne virus outbreaks have revealed the presence of similar virus strains over several years, indicating local overwintering of these viruses. However, it remains unclear how mosquito-borne viruses can persist in winter, when conditions are generally unfavourable for virus circulation. One of the presumed routes of virus persistence is via diapausing mosquitoes. Here, we set out to study whether arbovirus persistence of West Nile virus (WNV), Usutu virus (USUV) and Sindbis virus (SINV) occurs in diapausing mosquitoes in the Netherlands. To this end, mosquito collections were carried out in the winter of 2020 and 2021, in hibernacula located in two areas with previously observed WNV and/or USUV activity. In total, we collected 4200 mosquitoes belonging to four species (*Culex pipiens*, *Culiseta annulata*, *Anopheles maculipennis* s.l., and *Culex territans*), which were pooled in 490 monospecific pools. These pools were subjected to WNV-, USUV- and SINV-screening using a multiplex real-time RT-PCR assay. All mosquito pools tested negative for the presence of WNV, USUV and SINV RNA. Consequently, we did not find evidence of arbovirus persistence in diapausing mosquitoes in the Netherlands, even though USUV and WNV have re-appeared in birds and/or mosquitoes during the summer seasons of 2020–2022. Concluding, given the persistence of USUV and WNV in the Netherlands and SINV in other temperate regions, this study highlights the importance of further research on (alternative) arbovirus overwintering routes.

## 1 Introduction

In recent decades, several zoonotic mosquito-borne viruses, such as the flaviviruses West Nile virus (WNV) and Usutu virus (USUV) have become of increasing concern in temperate Europe. Interestingly, both viruses display similarities in their transmission cycles and partially overlap in geographical occurrence [1,2]. For both viruses, the omnipresent mosquito species *Culex pipiens* is recognized as the primary vector in North-Western Europe. This species is capable of transmitting both flaviviruses among avian hosts. In general, mammals are considered dead-end hosts, as they do not produce high enough viremia for mosquitoes to become infected after blood feeding [[2], [3], [4]]. Furthermore, *Cx. pipiens* is considered an important vector of the alphavirus Sindbis virus (SINV), of which the enzootic cycle is also maintained between mosquitoes and avian hosts [5].

Despite the similarities between WNV, USUV and SINV in terms of hosts and vectors, they differ with respect to their geographical occurrence and spread in both North- and North-Western Europe. WNV has been present in Europe for several decades [6]. However, until recently, the geographic distribution remained limited to Southern Europe and the Southern parts of Central- and Eastern Europe, but recently, the virus has spread as far northwards as Germany and the Netherlands [1]. In August 2020, WNV was detected in a common whitethroat (*Curruca communis*) in the municipality of Utrecht, the Netherlands. Shortly thereafter, mosquitoes belonging to the *Culex* genus were found infected with WNV after intensive bird- and mosquito sampling in the same region. These findings provided the first evidence of enzootic transmission of WNV in the country [7]. The detection of WNV in birds and mosquitoes was followed by the first human clinical cases of the virus after a rapid retrospective evaluation of suspected cases that was initiated immediately after the detection of WNV in birds and mosquitoes [8]. Although WNV was not detected in the Netherlands in the summer of 2021, in mid-September of 2022 a grey heron (*Ardea cinerea*) tested positive for the presence of WNV RNA in the province of North Holland, the Netherlands [9]. In other European countries, such as Germany and Hungary, WNV has been detected during summers of consecutive years, suggesting local persistence of the virus [10,11]. The first recorded finding of USUV in Europe occurred in 2001, in- and nearby Vienna, Austria [12]. Interestingly, retrospective analyses elucidated that USUV was likely already present in Italy as early as 1996 [13]. In 2016, USUV was detected for the first time in the Netherlands. The initial finding was characterized by a large-scale outbreak and high mortality among blackbirds [14]. After 2016, repeated outbreaks with genetically similar strains occurred in consecutive years, suggesting the possibility of local overwintering of the virus in either birds, mosquitoes or a yet unknown reservoir [[15], [16], [17]]. Another arbovirus relevant to Northern Europe is SINV. Recent phylogeographic research indicated that SINV was introduced in Northern Europe only once, several decades ago, after which it persisted in the initial area of introduction (Sweden) and spread to neighbouring countries [18]. However, seroprevalence screening has indicated presence of antibodies against SINV in wildlife in a wide range of European countries, of which some are adjacent to the Netherlands (such as Germany) [19]. Unlike WNV and USUV, SINV RNA and/or antibodies have not been detected in the Netherlands, and only one study investigated the presence of SINV in the Netherlands [20]. The current widespread seroprevalence of SINV highlights, however, the possibility of SINV being introduced to the Netherlands. Therefore, it is important to conduct SINV-surveillance, in vectors as well as in vertebrate hosts.

Despite the long term presence of these viruses in North- and North Western Europe, and the annual re-occurrence of genetically similar strains of USUV, the understanding of the underlying processes of mosquito-borne virus maintenance remains poorly understood [15]. In general, arbovirus maintenance in diapausing mosquitoes is considered one of the primary overwintering routes [21]. At northern latitudes, some mosquito species and subspecies are able to overcome unfavourable climatic conditions during winter as adults by entering diapause. Diapause is characterized by the cessation of ovariole development and limited activity. During this period, mosquitoes survive by utilizing lipid reserves that were built up prior to diapause. The primary vector of USUV and WNV, *Cx. pipiens*, consists of two morphologically indistinguishable biotypes, of which one (*Cx. pipiens pipiens*) enters diapause at the start of winter and the other (*Cx. pipiens molestus*) can remain active during winter [[22], [23], [24]]. Earlier studies have shown that during diapause and under favourable conditions, up to 70% of the *Cx. pipiens pipiens* population can survive [25], providing a large population of mosquitoes that can potentially carry arboviruses through winter.

In 2000, diapausing *Culex* mosquitoes, collected from a large number of hibernacula (i.e. abandoned houses, sewage water facilities, etc.) in New York, USA, tested positive for WNV RNA, albeit at very low percentages (<0.1%) [26]. The first findings of WNV RNA in diapausing mosquitoes in Europe were reported in 2017, when WNV RNA was detected in three pools (total no. of *Cx. pipiens* pools: 573) of diapausing *Cx. pipiens* mosquitoes collected in the Czech Republic [27]. Additional WNV-positive pools were found in diapausing *Cx. pipiens* mosquitoes from the same area several years later [28]. Additionally, evidence of WNV overwintering in diapausing *Cx. pipiens* mosquitoes was found in Germany in the winter of 2020/2021 [29]. Furthermore, examples of SINV persistence in diapausing mosquitoes has been observed in Sweden [30]. In general, the number of infected mosquitoes found in these studies is low, and as such, the relevance of diapausing mosquitoes as an arbovirus overwintering route remains unclear. Given the similarities between USUV and WNV mentioned earlier, it is possible that USUV maintenance in winter follows a similar route as WNV. However, USUV screening of diapausing mosquitoes only occurs sporadically, and thus far, evidence of overwintering is limited to a single anecdotal account from Austria [31]. The questions remain, therefore, whether 1) arbovirus overwintering occurs in diapausing mosquitoes in the Netherlands and 2) whether arbovirus overwintering in diapausing mosquitoes is a viable maintenance route in the Netherlands.

Here, we set out to study whether mosquito-borne viruses that have been detected during the summer season in the Netherlands persisted in diapausing, adult, female mosquitoes. To this end, we collected diapausing mosquitoes from their hibernacula in two areas where recently WNV- and/or USUV circulation was observed. Mosquito collections were carried out monthly during the winter of 2020/2021. Collected mosquitoes were identified morphologically and pooled based on species, sampling location and timepoint of sampling. Mosquito pools were subsequently subjected to molecular screening of WNV, USUV and SINV via real time RT-PCR.

## 2 Materials and methods

### 2.1 Mosquito collection

**Fig. 1:**
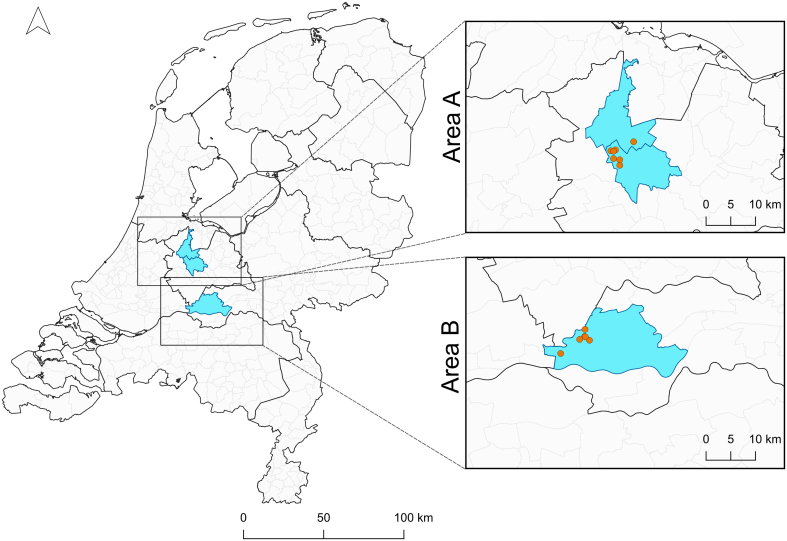
Locations of sampling areas. Area A: Area of USUV/WNV circulation in the summer of 2020. Area B: Area of USUV circulation in 2018. Orange dots indicate sampled hibernacula.

**Fig. 2:**
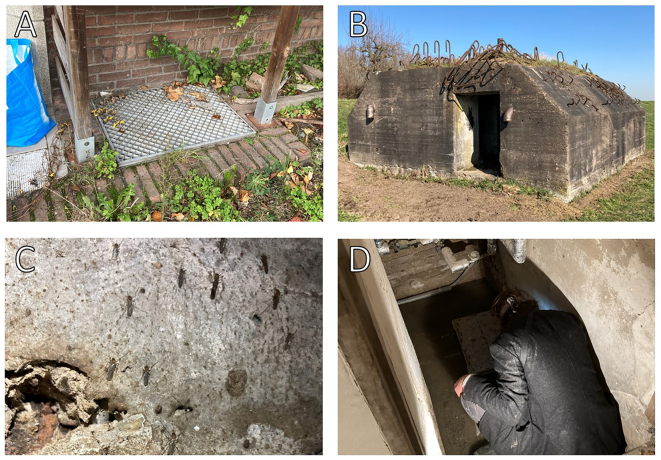
Sampling locations. A) A well in a residential area in the municipality of Utrecht, which housed several diapausing *Cx. pipiens* mosquitoes (Area A). B) A bunker of the New Hollandic Waterline (Area B). C) Diapausing *Cx. pipiens* mosquitoes on a wall of one of the bunkers of the New Hollandic Waterline. D) One of the researchers manually collecting diapausing mosquitoes from a cellar. Photo source: Rody Blom.

In the winter of 2020/2021, mosquito collections were carried out monthly in two study areas (Area A and Area B). Area A comprises the municipalities of Stichtse Vecht and Utrecht, the Netherlands (Fig. 1). This area was selected as it was nearby the location where enzootic circulation of WNV was detected in 2020 and USUV was detected in earlier summers and the summers of 2021 and 2022 [7,[15], [16], [17]]. In Area A, seven hibernacula were sampled, which consisted of human-made structures, including (bat) cellars, wells and chicken pens (Fig. 2). Mosquitoes were sampled from this area from October 2020 until March 2021. Area B is located in the municipality of West-Betuwe, the Netherlands (Fig. 1). From December 2020 until April 2021, monthly mosquito sampling at Area B was conducted in six late 19th century bunkers of the New Hollandic Waterline (Fig. 2). These bunkers are well documented hibernacula for a range of mosquito species, as well as other insects and bats [25,32]. In late July 2018, one USUV RNA-positive Eurasian blackbird (*Turdus merula*) was collected from this area [15]. All mosquito collections were carried out using manual- and/or automatic aspiration, with the primary focus being the collection of *Cx. pipiens* mosquitoes. After collection, mosquitoes were transported to the Laboratory of Entomology, Wageningen University and Research, Wageningen, the Netherlands. Mosquitoes were stored for up to 15 months at −20 °C until identification and further processing.

### 2.2 Morphological identification and pooling

Mosquito identification was performed based on morphological characteristics as described by Becker et al. [33]. After identification, monospecific mosquito pools with a maximum of ten mosquitoes per pool were made per location and time point of sampling. This resulted in 129 pools of *Cx. pipiens*, 5 pools of *Culiseta annulata*, 7 pools of *Anopheles maculipennis* s.l. (sensu lato) and 3 pools of *Culex territans* for Area A and 211 pools of *Cx. pipiens*, 66 pools of *Cs. annulata*, 45 pools of *An. maculipennis* s.l. and 24 pools of *Cx. territans* for Area B. All mosquito identifications and pooling were conducted on a cold plate in order to maintain a cold chain. Descriptive statistics of mosquito catches were calculated using Microsoft Excel.

### 2.3 Sample processing

Mosquito pools were homogenized in 1 ml cold medium (DMEM with 4.5 g/l glucose (Lonza, Verviers, Belgium), NaHCO3 (0.075%, Lonza, Verviers, Belgium), Hepes buffered saline (17 mM, Lonza, Verviers, Belgium), penicillin/streptomycin (1000 U/ml, Lonza, Verviers, Belgium), amphotericin (0.0125 mg/ml, in house made)) with one 1/4″ ceramic sphere (MP Biomedicals, Solon OH, USA) using the FastPrep-24 5G Homogenizer MP Biomedicals (30″ 5 m/s). Mosquito pools were placed at +4 °C for at least 5 min before spinning down the samples for 1 min at 13,000 g.

### 2.4 Real-time RT-PCR

First, 60 μl of supernatant was added to 90 μl lysis buffer (MagNA Pure 96 External Lysis Buffer, Roche LifeScience, Basel, Switzerland). Phocine Distemper Virus (PDV) was added and used as internal control as described previously [34]. Subsequently, 50 μl of Ampure beads was added and incubated for 15 min. Samples were washed 3 times with 70% ethanol using a DynaMag 96 side magnet (Invitrogen/Thermo Fisher, Waltham, MA, USA) and eluted in 50 μl water. Samples were tested using real-time RT-PCR in duplex reactions; WNV [7] combined with PDV [35] and USUV [36] combined with SINV [37] (Table 1). An input of 8 μl RNA was used in a 20 μl reaction using TaqMan Fast Virus 1-Step Master Mix (Thermo Fisher, Waltham, MA, USA). RT-PCRs were performed using the LightCyler 480 (Roche LifeScience, Basel, Switzerland). Fit point analysis was used to determine the Ct values, thereby the cut-off threshold was set manually above the background signals of the negative controls. The following thermal cycling program was used: 50 °C for 5 min, 95 °C for 20 s followed by 45 cycles of 95 °C for 3 s and 60 °C for 30 s.

**Table 1:** Primer- and probe sequences used for screening of WNV, USUV and SINV.

Target	Forward primer5′ – 3′	Reverse primer5′ – 3′	Probe 5′ – 3′
			
WNV C	CCACCGGAAGTTGAGTAGACG	TTTGGTCACCCAGTCCTCCT	TGCTGCTGCCTGCGGCTCAACCC
USUV NS5	CAAAGCTGGACAGACATCCCTTAC	CGTAGATGTTTTCAGCCCACGT	AAGACATATGGTGTGGAAGCCTGATAGGCA
SINV NSP1	GGTTCCTACCACAGCGACGAT	ATACTGGTGCTCGGAAAACATTCT	TTGGACATAGGCAGCGCACCGG
PDV HA	CGGGTGCCTTTTACAAGAAC	TTCTTTCCTCAACCTCGTCC	ATGCAAGGGCCAATT

## 3 Results

In total, 4200 mosquitoes were collected over the course of seven months (October until April). Mosquito collections from Area A resulted in a total number of 1225 female mosquitoes of four different species. In total, 1178 *Cx. pipiens* (96.2%), 17 *C*s. *annulata* (1.4%), 25 *An*. *maculipennis* s.l. (2.0%) and five *Cx. territans* (0.4%) were collected. From Area B, 2975 female mosquitoes were collected, comprising four species: 1975 *Cx. pipiens* (66.4%), 509 *Cs. annulata* (17.1%), 361 *An. maculipennis* s.l. (12.1%) and 135 *Cx. territans* (4.4%) (Table 2). All 490 mosquito pools tested negative for the presence of WNV-, USUV- and SINV RNA.

**Table 2:** Monthly- and total number of mosquitoes collected from hibernacula in the municipalities of Utrecht and Stichtse Vecht (Area A) and West-Betuwe (Area B), the Netherlands during the winter of late 2020 and early 2021.

	Species	Oct	Nov	Dec	Jan	Feb	Mar	Apr	Total
Area A	*Culex pipiens*	410	353	218	130	43	24	–	1178
	*Culiseta annulata*	0	7	5	0	0	5	–	17
	*Anopheles maculipennis* s.l.	0	12	4	0	3	6	–	25
	*Culex territans*	1	1	3	0	0	0	–	5
Area B	*Culex pipiens*	–	–	906	403	456	208	2	1975
	*Culiseta annulata*	–	–	186	137	135	51	0	509
	*Anopheles maculipennis* s.l.	–	–	150	133	NA⁎	53	25	361
	*Culex territans*	–	–	52	31	32	13	2	135

⁎ *Anopheles maculipennis* s.l. were removed from the analysis for the use in another study [32].

## 4 Discussion

In this study, we aimed to assess whether mosquito-borne flaviviruses, which caused outbreaks in birds during summer, overwinter in diapausing mosquitoes. None of the mosquito pools screened for arboviruses tested positive for WNV-, USUV- or SINV RNA. While the absence of evidence of viruses in diapausing mosquitoes does not imply the absence of this overwintering route, it does highlight the importance of investigating other routes of virus overwintering, such as possible maintenance of viruses within winter-active mosquito communities and bird-to-bird transmission.

Vertical transmission (transmission from parent mosquito to offspring) is considered to be a prime driving mechanism for WNV overwintering. Prior to diapause, environmental cues (i.e. shortening daylight, decrease in temperature) prompt the last summer/early autumn-generation of *Cx. pipiens pipiens* larvae to enter diapause as adult. In general, these newly emerged *Cx. pipiens pipiens* adults mate and feed solely on nectar, which they use to build up fat reserves [38]. It is therefore likely that diapausing mosquitoes that are infected with a virus, became infected via vertical transmission instead of feeding from an infected host. Although unlikely, it is possible that mosquitoes take up an infectious bloodmeal prior to diapause without utilizing it for oogenesis (gonotrophic dissociation), which can serve as another route for mosquitoes to become infected prior to diapause, in addition to vertical transmission [39,40]. However, given the very limited evidence of gonotrophic dissociation occurring, vertical transmission seems the more likely route of WNV persistence in diapausing mosquitoes. Several laboratory studies have shown that vertical transmission of WNV is possible for various mosquito species, although rates generally remain low (minimum filial infection rate = 0–6.9 per 1000 mosquitoes tested) [[40], [41], [42], [43]]. Furthermore, immature stages of *Cx. pipiens* mosquitoes (eggs and pupae) have been collected in the field and subsequently tested positive for WNV [44,45]. Despite the high survival rate (approximately 70%) of diapausing *Cx. pipiens pipiens* mosquitoes, the low infection rates resulting from vertical transmission suggest that the process of arbovirus overwintering in diapausing mosquitoes only occurs sporadically [25].

The probability of flavivirus detection in diapausing mosquitoes is further complicated by the fact that a very low proportion of mosquitoes is usually infected in outbreak situations, and that the circulation of WNV and USUV may be patchy in countries/areas like the Netherlands. Minimum infection rates (MIRs (= number of positive pools/total number of tested specimens*1000)) of USUV in overwintering mosquitoes remain unknown as they have never been reported, whereas in previous studies from the US, Germany and the Czech Republic, MIRs of WNV in diapausing mosquitoes ranged between 0.04 and 1.93 [26,27,29,46]. In addition, in some cases exceptionally high MIRs have been calculated, such as in the Czech Republic in 2019 (MIR = 4.27, all mosquito species) [28]. Assuming persistence of WNV in our study area, a minimum number of expected positive pools in our study would range between 0.2 and 8.1 for all mosquito species and 0.1 and 6.1 for *Cx. pipiens* specifically, based on the MIRs calculated in previous studies (excluding Czech Republic in 2019). In Europe, the lowest number of collected mosquitoes that resulted in a WNV RNA-positive pool was 6101 in a study from Germany [29]. Assuming that only one mosquito in the WNV RNA-positive pool was infected, an infection rate of 0.016% can be calculated. Assuming similar infection rates in the Netherlands, a total number of 0.67 WNV RNA-positive mosquitoes could be expected in our samples. This indicates that an increased sampling effort may result in WNV-positive mosquito pools. It should be noted, however, that despite the re-appearance of WNV in 2022, no WNV RNA-positive mosquitoes or birds were detected in 2021 [9]. USUV, on the other hand, was observed in every year of the study, which we expected to be mirrored in positive mosquito pools in winter. Furthermore, we sampled the vast majority of available anthropogenic overwintering sites in our study areas, thus maximizing the number of collected mosquitoes. Given the number of mosquitoes screened for arboviruses in our study and the absence of positive samples, we conclude that persistence of arboviruses in our study areas is either not maintained in diapausing mosquitoes, or at rates that were below detection based on our sampling effort.

Alternatively, arboviruses could persist in winter-active *Cx. pipiens* mosquitoes, although in general, virus replication in mosquitoes is low under winter temperatures and increases at higher temperatures [47,48]. As stated earlier, individuals belonging to the *Cx. pipiens molestus* biotype remain active and occupy warmer microclimates indoors throughout winter [22]. Collection of winter-active vector species from these indoor habitats and screening them for the presence of arboviruses may prove useful. The habitats occupied by *Cx. pipiens molestus* in winter mostly consist of private property and as such, mosquito collections from these locations are more difficult than from more publicly accessible sites as sampled in current study. However, citizen science is a valuable tool in collecting mosquitoes from otherwise hard-to-reach places, as demonstrated by Vogels et al. (2015)[22]. In addition, citizen science can be used to collect samples from the domestic environment at a high spatial coverage. As of yet, there are no published studies on the presence of arboviruses in mosquitoes collected via citizen science in winter, but we have initiated follow-up studies to address this topic.

The role other overwintering mosquito species (i.e. *Cx. torrentium*, *Cx. territans*, *Culex modestus*, *Cs. annulata*, and *An. maculipennis* s.l.) might play in the persistence of flaviviruses throughout winter remains unclear. To the best of our knowledge, none of these species have been tested positive for WNV or USUV in winter in earlier studies. The vast majority of mosquitoes collected in our study (75%) were *Cx. pipiens,* the primary vector of WNV and USUV in North-Western Europe. Species within the *An. maculipennis* species complex and *Cx. territans* are not considered to play an important role in WNV, USUV and/or SINV transmission. *Culiseta annulata* has not been associated with WNV transmission, although USUV has been isolated from field collected *Cs. annulata* in Italy in 2011 [49]. *Culex modestus*, a known WNV vector that overwinters as an adult, was not found in the hibernacula sampled in this study [28,50,51]. Of the mosquitoes collected in our study, only *Cx. pipiens* is considered a competent vector for WNV and USUV [52,53]. It should be noted, however, that vector competence for USUV and WNV has never been studied for most of the aforementioned species. In order to assess the role these mosquitoes may play in arbovirus overwintering, more research is needed on vector competence and infections in wild mosquitoes.

Given the lack of evidence of virus overwintering in diapausing mosquitoes in the Netherlands, it is necessary to conduct research on potential alternative routes of virus maintenance in winter. As observed in other countries, repeated outbreaks of WNV and/or USUV may in part be explained by the occurrence of virus reintroductions and/or maintenance of the viruses without vector activity. Repeated introductions of WNV and USUV may occur via infected migratory birds, despite the lack of conclusive evidence [31,54]. Additionally, bird-to-bird transmission in winter has been observed for both USUV [55] and WNV [56], and as such, this also remains a potential overwintering mechanism for WNV and USUV. Finally, the role of other vectors and non-avian reservoir hosts (such as mammals) remains mostly unexplored, and additional research might provide new insights in arbovirus overwintering mechanisms.

Concluding, the underlying mechanisms of repeated arbovirus outbreaks in the Netherlands remain enigmatic. Given the evidence from other countries, arbovirus maintenance in diapausing mosquitoes seems likely, but we did not find evidence to support this hypothesis. To further improve predictions on mosquito borne disease outbreaks and to understand the role of mosquitoes in transmitting pathogens from animals to humans, it remains necessary to explore other potential routes of arbovirus persistence, whilst also continuing research on arbovirus overwintering in mosquitoes. Furthermore, while no evidence of the presence of SINV was found, continued surveillance of SINV is needed, especially given its current geographical spread.

## Authors' contribution

**Rody Blom:** Conceptualization, Investigation, Writing - original draft, Visualization. **Maarten J.J. Schrama:** Conceptualization, Investigation, Writing - review & editing. **Jeroen Spitzen:** Investigation, Writing - review & editing. **Babette F.M. Weller:** Investigation, Writing - review & editing. **Anne van der Linden:** Investigation, Writing - review & editing. **Reina S. Sikkema:** Investigation, Writing - review & editing. **Marion P.G. Koopmans:** Funding acquisition, Writing - review & editing. **Constantianus J.M. Koenraadt:** Conceptualization, Investigation, Writing - review & editing.

All authors read and approved the final manuscript for publication.

## Declaration of Competing Interest

The authors declare that they have no known competing financial interests or personal relationships that could have appeared to influence the work reported in this paper.

## Data Availability

Data will be made available on request.
